# Frequency and pattern of dyslipidemia and its association with other risk factors among Type-2 Diabetics

**DOI:** 10.12669/pjms.41.2.10264

**Published:** 2025-02

**Authors:** Alia Ali Muhammad, Manal Afzaal, Mehak Joher Khan, Amena Moazzam Baig, Muhammad Aasim

**Affiliations:** 1 Alia Ali Muhammad, FCPS Assistant Professor, Department of Medicine, Shaikh Zayed Postgraduate Medical Institute, Lahore, Pakistan; 2 Manal Afzal, FCPS Assistant Professor, Department of Medicine, Shaikh Zayed Postgraduate Medical Institute, Lahore, Pakistan; 3 Mehak Joher Khan Second Professional Medical Student, FMH College of Medicine and Dentistry, Lahore, Pakistan; 4 Amena Moazzam Baig, FCPS, FRCP Assistant Professor, Department of Endocrinology and Metabolism, Services Institute of Medical Sciences, Lahore, Pakistan; 5 Muhammad Aasim Statistician, NHRC, NIH-HRI Research Center, Shaikh Zayed Postgraduate Medical Institute, Lahore, Pakistan

**Keywords:** Dyslipidemia, Obesity, Smoking, Type-2 DM

## Abstract

**Objective:**

To determine the frequency and pattern of dyslipidemia and its association with other risk factors among Type-2 diabetics.

**Methods:**

This cross-sectional study was conducted in Diabetic Clinic of Shaikh Zayed Postgraduate Medical Institute Lahore from January-October 2023. Type-2 diabetics regardless of age were evaluated by fasting lipid profile and were further evaluated by BMI, HbA1c and smoking. Exclusion criteria was patients taking lipid lowering drugs and all other causes of secondary dyslipidemias other than diabetes. Data for normally distributed quantitative variables was presented by Mean±SD, and for skewed measures were presented by using median (IQR). Dyslipidemia was described for each trait by using frequency and percentages.

**Results:**

A total of two hundred and twelve subjects were included in the study with the mean age of 53.4±9.1 years. About 96.6% patients were found to have different patterns of dyslipidemia that also showed positive association with smoking, uncontrolled HbA1c and obesity (p < 0.001).

**Conclusion:**

There is very high prevalence of dyslipidemia among Type-2 diabetics especially those who are smokers, with poor glycemic control and overweight/obese. Early screening of patients for dyslipidemia with lifestyle modifications and treatment of dyslipidemia can reduce risk of CVD.

## INTRODUCTION

Diabetes is a global public health problem, affected nearly half a billion people (463 million) in the world. Around five million adult people died from diabetes and diabetes related complications in 2019.^1^ The frequency of diabetes mellitus is increasing many folds in South Asian population due to the high degree of genetic predisposition and high susceptibility to environmental insults characterized by a high BMI, upper body adiposity, a high body fat percentage and a high level of insulin resistance.^2^ A very common metabolic abnormality associated with diabetes is dyslipidemia, which is characterized by a spectrum of quantitative and qualitative changes in lipids and lipoproteins.^3^

Dyslipidemia is one of the major risk factors for cardiovascular diseases in diabetes mellitus. The risk attributable to cholesterol increases with increasing levels in diabetics as compared to non-diabetic subjects.^4^ In diabetes mellitus, lipid abnormalities are more prevalent because major key enzymes and lipid metabolism pathway are affected due to deficiency of insulin production and secretion. The etiology leading to hypertriglyceridemia in T2DM directly relates to insulin resistance and hyperglycemia, which results in an overproduction of triglyceride rich lipoproteins from the liver, decreased clearance of triglyceride rich lipoproteins, and in some cases, an altered postprandial lipoprotein metabolism.^5^

Early detection and treatment of dyslipidemias in Type-2 diabetes mellitus can prevent risk for atherogenic cardiovascular disorders.^6^ High triglycerides, low high density lipoprotein (HDL cholesterol) and increase low density lipoprotein (LDL cholesterol) are characteristic features of diabetic dyslipidemia. These metabolic dysregulators like dyslipidemia and obesity along with other lifestyle factors are known to be associated with Type-2 diabetes mellitus characterized by hyperglycemia.^7^

Global burden of dyslipidemia in diabetic patients is continuously increasing due to increased consumption of unhealthy diets, reduced physical activity, and urbanization as well as obesity.^8^ The major risk factors for dyslipidemia were hypertension, high body mass index, aging, high fasting blood sugar, physical inactivity and longer duration of diabetes mellitus.^9^

Dyslipidemia is the most common and clinically relevant metabolic abnormality in patients with Type-2 diabetes mellitus, contributing to an increased cardiovascular disease risk. Knowledge of the patterns and prevalence of dyslipidemia in untreated diabetic patients represents critical input into the natural history of lipid derangements uninfluenced by pharmacologic interventions. The present study aims at determining the frequency and distribution of dyslipidemia traits, including LDL, HDL, triglycerides, and total cholesterol, and their association with key risk factors such as obesity, glycemic control, and smoking status. These findings will be new addition of knowledge about patterns of dyslipidemia in Pakistan population (very limited data available) and will help in guiding early screening and non-pharmacological intervention strategies for reduction of cardiovascular risk in the vulnerable population.

## METHODS

A cross-sectional study was performed at the Endocrinal/diabetic clinic of department of medicine, Shaikh Zayed Postgraduate Institute, Lahore, Pakistan.

### Ethical approval:

Institutional ethical review approval (ERC# SZMC/TERC//401/2022, dated August 25,2022) was obtained before the start of study.

A total of 212 Type-2 diabetic patients who attended the diabetic outdoor clinic from January- October 2023, were included in the study. Patients were assessed for dyslipidemia by measuring their fasting lipid profile. Five ml (5ml) of venous blood samples were collected from fasting diabetic patients and serum was separated by centrifuging the blood samples at 4000rpm for four minutes for lipid profile analysis including Total cholesterol, Triglycerides, HDL cholesterol, LDL cholesterol. Dyslipidemia is defined as Total cholesterol ≥200mg/dl, Triglycerides ≥150mg/dl, HDL Cholesterol <60mg/dl and LDL Cholesterol ≥100mg/dl.

The target HbA1c was determined to be ≤7% according to American Diabetic Association (ADA) guidelines. Thus, glycemic control was considered well controlled with HbA1c levels ≤7%, while poor controlled DM with HbA1c levels of >7%.^10^ BMI was computed as weight in kilograms divided by the square of height in meter and characterized as normal 18.5-24.9Kg/m^2^, overweight 25-29.9Kg/m^2^ and obese ≥30Kg/m^2^.

### Inclusion criteria:

All Type-2 diabetic patients seen as outpatients, on any mode of treatment for diabetes, were included in the study.

### Exclusion criteria:

Diabetic patients taking lipid lowering drugs, who were pregnant and who had a known history of cardiac problems, chronic liver and renal diseases, and secondary dyslipidemias were excluded by normal LFTs, RFTs, Thyroid function tests. Patients were provided with informed consent and ethical clearance from IRB was taken.

### Sample Size:

These 212 Types-2 diabetic patients were estimated by using 95% confidence level, 5% margin of error with expected frequency of dyslipidemia 83.5% among diabetic patients.^8^

### Statistical analysis:

Data was entered and analyzed by using SPSS version 20.0. Data for normally distributed quantitative variables was presented by Mean±SD, and for skewed measures was presented by using median (IQR). Dyslipidemia was described for each trait by using frequency and percentages. Association of dyslipidemia for each trait and for number of traits with, diabetes control, body mass index and smoking status were tested by using chi-square test. A p-value ≤ 0.05 was considered statistically significant.

## RESULTS

The study included 212 diabetic cases, with mean age of 53.4±9.1 years. Among these 132(62.3%) were males, 168(79.2%) had family history of diabetes, 61(28.8%) were smokers while 86(40.6%) also had hypertension. The median BMI was 27.0(24.0 – 30.0) kg/m^2^, HbA1c was 7.0(6.7 – 8.0), Total cholesterol was 214.5(186.5 – 250.0) mg/dl, Triglyceride levels of 179(150 – 200) mg/dl, and LDL and HDL cholesterols were recorded 110(99 – 136) and 43(40- 46) mg/dl respectively.

**Fig.1:**
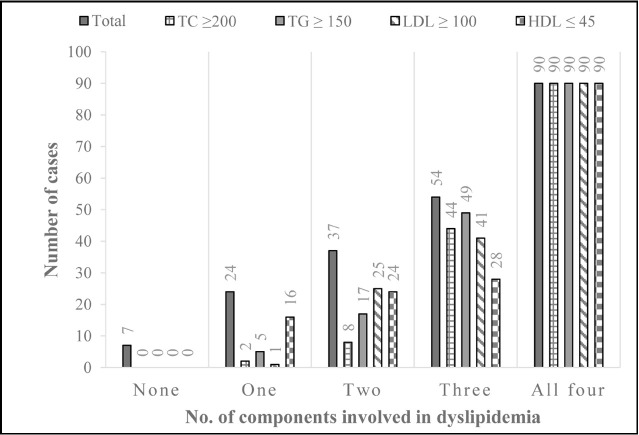
Total components involved in dyslipidemia in relation to individual components.

There were only seven (3.3%) cases who had no factor of dyslipidemia positive. The one trait of dyslipidemia was found among 24 (11.3%) cases and the most common single trait was the low HDL level. Among 37 (17.5%) of the cases having two of the traits positive, the low HDL level and high LDL level were most common. Among these cases, 54 had three traits of dyslipidemia and among them triglyceride was most common followed by total cholesterol, LDL cholesterol and HDL cholesterol. In total 90 (42.5%) cases had complete dyslipidemia picture with all four traits present, (Fig.1).

The diabetic control had a very strong association with dyslipidemia. Among total cases, 114 (53.8%) cases were found to have good diabetic control. The dyslipidemia with respect to total cholesterol was present in 88 (89.8%) of poor diabetic control (PDC) patients as compared to 49.1% among good diabetic control (GDC). The triglyceride was higher among 96.9% of PDC as compared to 57.9% among GDC. Similarly, the difference for LDL cholesterol was also above 23.0% between PDC and GDC patients. All seven cases free of any kind of dyslipidemia were found among GDC and the prevalence of number of traits of dyslipidemia increased significantly among PDC as compared to GDC. The only factor which was not found significantly different among GDC and PDC groups was HDL cholesterol level, which was found lower than 45 mg/dl in 76.3% of GDC and 72.4% of PDC, (Table-I).

**Table-I:** Dyslipidemia in relation to Diabetes control among diabetic patients.

		HbA1c						P-value
		≤ 7.0 Good control (n = 114)		> 7.0 Poor control (n = 98)		Total (n = 212)		
		n	%	n	%	n	%	
Cholesterol	≥ 200	56	49.1	88	89.8	144	67.9	<0.001
	< 200	58	50.9	10	10.2	68	32.1	
Triglycerides	≥ 150	66	57.9	95	96.9	161	75.9	<0.001
	< 150	48	42.1	3	3.1	51	24.1	
LDL cholesterol	≥ 100	72	63.2	85	86.7	157	74.1	<0.001
	< 100	42	36.8	13	13.3	55	25.9	
HDL Cholesterol	≤ 45	87	76.3	71	72.4	158	74.5	0.627
	> 45	27	23.7	27	27.6	54	25.5	
At least one trait of dyslipidemia	Yes	107	93.9	98	100.0	205	96.7	0.016
	No	7	6.1	0	0.0	7	3.3	
No of components of dyslipidemia	None	7	6.1	0	0.0	7	3.3	<0.001
	One	21	18.4	3	3.1	24	11.3	
	Two	29	25.4	8	8.2	37	17.5	
	Three	26	22.8	28	28.6	54	25.5	
	Four	31	27.2	59	60.2	90	42.5	

The obesity is also considered one of the reasons of dyslipidemia as well. There were 57(26.9%) of the cases who were obese (BMI≥30), 82(38.7%) were overweight and others were normal on BMI. When dyslipidemia was checked in relation to these three categories of BMI, the total cholesterol and triglycerides were highly significantly associated with BMI with p-values <0.001, and HDL had significant association with p-value 0.009. It was interesting to note that there were 81.7% cases among overweight with TC≥200 and obese had 77.2% in comparison to 45.2% among normal weight diabetics. For HDL cholesterol, those who were obese had 59.6% of cases with HDL≤45 mg/dl, while among overweight there were 82.9% and among normal weight there were 76.7% cases. Cases with one and two traits of dyslipidemia were more common among normal weight cases i.e., 17.8% and 30.1% as compared to 9.8% and 6.1% among overweight and 5.3% and 17.5% among obese. On the other hand, four traits were most common among overweight with 59.8% cases followed by 45.6% among obese and 20.5% among normal weight cases, (Table-II).

**Table-II:** Dyslipidemia in relation to body mass index among diabetic patients.

		BMI								P-value
		< 25.0 (n = 73)		25.0 - 29.9 (n = 82)		≥ 30.0 (n = 57)		Total (n = 212)		
		n	%	n	%	n	%	n	%	
Cholesterol	≥ 200	33	45.2	67	81.7	44	77.2	144	67.9	<0.001
	< 200	40	54.8	15	18.3	13	22.8	68	32.1	
Triglycerides	≥ 150	41	56.2	70	85.4	50	87.7	161	75.9	<0.001
	< 150	32	43.8	12	14.6	7	12.3	51	24.1	
LDL cholesterol	≥ 100	47	64.4	66	80.5	44	77.2	157	74.1	0.064
	< 100	26	35.6	16	19.5	13	22.8	55	25.9	
HDL Cholesterol	≤ 45	56	76.7	68	82.9	34	59.6	158	74.5	0.009
	> 45	17	23.3	14	17.1	23	40.4	54	25.5	
At least one trait of dyslipidemia	Yes	70	95.9	81	98.8	54	94.7	205	96.7	0.336
	No	3	4.1	1	1.2	3	5.3	7	3.3	
No of components of dyslipidemia	None	3	4.1	1	1.2	3	5.3	7	3.3	<0.001
	One	13	17.8	8	9.8	3	5.3	24	11.3	
	Two	22	30.1	5	6.1	10	17.5	37	17.5	
	Three	20	27.4	19	23.2	15	26.3	54	25.5	
	Four	15	20.5	49	59.8	26	45.6	90	42.5	

It was observed that the total cholesterol and traits of dyslipidemia were significantly associated with smoking having p-values 0.003 and <0.001 respectively, while triglycerides had a close to significant association with p-value 0.066. There were 83.6% cases among smokers who had TC≥200 mg/dl as compared to 61.6% among non-smokers. The high triglycerides were observed among 85.2% of smokers as compared to 72.2% among non-smokers. The two traits of dyslipidemia were more common (23.8%) among non-smokers as compared to smokers (1.6%), while four traits of dyslipidemia were found present among 62.3% of smokers as compared to 34.4% of non-smokers, (Table-III).

**Table-III:** Dyslipidemia in relation to smoking status of diabetic patients.

		Smoker						P-value
		Yes		No		Total		
		n	%	n	%	n	%	
Cholesterol	≥ 200	51	83.6	93	61.6	144	67.9	0.003
	< 200	10	16.4	58	38.4	68	32.1	
Triglycerides	≥ 150	52	85.2	109	72.2	161	75.9	0.066
	< 150	9	14.8	42	27.8	51	24.1	
LDL cholesterol	≥ 100	50	82.0	107	70.9	157	74.1	0.134
	< 100	11	18.0	44	29.1	55	25.9	
HDL Cholesterol	≤ 45	47	77.0	111	73.5	158	74.5	0.718
	> 45	14	23.0	40	26.5	54	25.5	
At least one trait of dyslipidemia	Yes	59	96.7	146	96.7	205	96.7	1.000
	No	2	3.3	5	3.3	7	3.3	
No of components of dyslipidemia	None	2	3.3	5	3.3	7	3.3	<0.001
	One	7	11.5	17	11.3	24	11.3	
	Two	1	1.6	36	23.8	37	17.5	
	Three	13	21.3	41	27.2	54	25.5	
	Four	38	62.3	52	34.4	90	42.5	

## DISCUSSION

In our study, we observed very high frequency of dyslipidemia among Type-2 diabetic patients. Out of 212 patients, 205 (97%) were having dyslipidemia either as a single trait or more than two traits present, while 90% patients (42.5%) had complete dyslipidemia with all four traits (TC, TG, LDL, HDL) present. Similarly, study by Al Quran TM et al.^11^ showed frequency of dyslipidemia 97.4% just similar to our finding whereas regarding most common pattern observed in our study was high triglycerides, p value=<0.001, low HDL-C, p value=<0.627 as compared to Al Quran et al that showed the most common pattern observed was high LDL-C (62.1%) and low HDL-C (66.2%) respectively.

Another recent study from Nigeria by Beatrice OB et al^12^ showed 69.3% frequency of dyslipidemia that is quite lower than our study and most common pattern of dyslipidemia as high TGs and High LDL-C (41.0%) that is similar to our finding. This high prevalence is also shown by many other studies conducted at different countries.^13^-^16^

Our study also observed positive association between diabetic smokers and dyslipidemia showing p-value of 0.33. Similarly, Ahmmed MS et al. also showed increase frequency of dyslipidemia among diabetic smokers as compared to non-smokers, P- value 0.03.^17^ Al- Quran et al also showed that smokers were more likely to develop dyslipidemias than non-smokers, P-value 0.001.^11^ In our study, poor glycemic control was an independent risk factor for developing dyslipidemia (high LDL-C, high total Cholesterol and high TGs) among Type-2 diabetics. Dyslipidemia with respect to total cholesterol was present in 89.8% of patients with poor glycemic control (HbA1c > 6.5) 46.2% of patients with poor glycemic control had one or more traits of dyslipidemia.

Similarly, study by Al Quran also showed positive association between poor glycemic control and dyslipidemia (p-value<0.001)^11^ Jia hang Li et al also showed poor glycemic control (HbA1c >7%) as a major factor predicting dyslipidemia among Type-2 diabetics. (p-value <0.0001).^18^ Dyslipidemia has been suggested to have a linear relationship with HbA1c as showed by many studies.^19^-^22^ Dana H et al.^23^ also showed that patients with HbA1c (7-8%) were two times more likely to have a high cholesterol level (≥200mg/dl) as compared with those with HbA1c level ≤7% (p- value=0.021).^21^

Moreover, researchers also have examined that obesity increases lipid profile and significant impact on HbA1c level, as a result of insulin inactivity.^17^,^24^,^25^ In our study, obesity is also considered as one of the reasons for dyslipidemia as dyslipidemia was significantly associated with BMI (P value <0.001). Ahmmed MS et al also revealed that obesity was significantly associated with dyslipidemia in Type-2 diabetic patients (p<0.001).^17^ Similarly, Zikiria et al also showed that as compared to normal weight subjects, obese Type-2 diabetics had 27.3% increase in the prevalence of dyslipidemia.^26^ Narindrarangkura P et al also showed 90.19% of dyslipidemia in patients with higher BMI (25.0-29.9kg/m2), p-value of <0.001.^27^

Similarly, Mehta et al showed that dyslipidemia was more prevalent in patients having higher BMI, and high LDL (93.5%) was the most prevalent lipid abnormality followed by mixed dyslipidemia (89.7%).^28^ Early recognition of dyslipidemias, by screening lipid profile, and its associated risks like poor glycemic control and obesity should be managed as dyslipidemias are increasing at alarming figures in our country.

Extensive studies are warranted to further determine and highlight other associated risk factors like hypertension and cardiovascular risks. A longitudinal study would provide deeper insights and we recommend for future research.

### Limitations:

A longitudinal study would provide deeper insights and after validation these patterns may come up as screening option for Type-2 diabetes as well. Data on additional metabolic parameters like blood pressure, kidney function, the lack of smoking intensity data, and the exclusion of patients on lipid-lowering drugs, was not collected. We acknowledge this limitation and recommend exploring these factors in future studies.

## CONCLUSION

In summary, our study concluded that dyslipidemia is highly prevalent among Type-2 diabetics especially who are overweight/obese, smokers and having unacceptable glycemic control. Thus, adequate knowledge among public, especially diabetics, to maintain healthy weight, dietary modifications in the form of more fiber and less fats, regular exercise, good glycemic control would lead to less burden on health care system that is already crippled with diabetes burden.

The primary healthcare facilities should execute regular follow up, monitoring, proper advice, and intervention programs to reduce the prevalence of dyslipidemias among Type-2 diabetes patients thus leading to less burden on patients to be treated for dyslipidemias who are already economically burden with diabetes treatment.

### Author’s contributions:

**AAM, MJK and MA:** Conception and design, data acquisition. analysis and interpretation.

All authors have participated in the drafting of the manuscript.

**ABM and MA:** Critical Revision,

All authors have read and approved the final version of the manuscript and are accountable for the integrity of the study.

## References

[1] Int Dia Federation (2019). IDF Diabetes Atlas Ninth edition.

[2] Uttra KM (2011). Lipid Profile of patients with diabetes mellitus. A multidisciplinary study. World Appl Sci J.

[3] Liya Wu, Klaus G (2014). Diabetic Dyslipidemia. J Metabol.

[4] Yoshino G, Hirano T, Kazumi T (1996). Dyslipidemia in diabetes mellitus. Diabetes Res Clin Pract.

[5] Taskinen MR (2002). Diabetic dyslipidemia. Atheroscles.

[6] Dixit AK, Dey R, Suresh A, Chaudhuri S, Panda AK, Mitra A (2014). The prevalence of dyslipidemia in patients with diabetes mellitus of ayurveda Hospital. J Diabetes Metab Disord.

[7] American Diabetes association (2009). Diagnosis and classification of diabetes mellitus. Diabetic care.

[8] Abhishek Gupta, Vinod Kumar Tyagi, Sunil Kumar Virmani (2015). Evaluation of pattern of dyslipidemia and its association with obesity in Type-2 diabetes mellitus. Inter J Cont Med Res.

[9] Ghouth ASB, Ba-Karman AA, Alaidroos HA, Alajely MH, Bin-Talib MH, Jadnan RSB (2019). Prevalence and Patterns of Dyslipidemia among Type-2 Diabetes Mellitus Patients in Mukalla city, Yemen, in 2017. J Community Med Public Health Care.

[10] American Diabetes Association (2020). Summary of revisions:standards of medical care in diabetes-2020. Diabetes Care.

[11] Al Quran TM, Bataineh ZA, Al-Mistarehi AH, Zein Alaabdin AM, Allan H, Al Qura'an A (2022). Prevalence and Pattern of Dyslipidemia and Its Associated Factors Among Patients with Type-2 Diabetes Mellitus in Jordan:A Cross-Sectional Study. Int J Gen Med.

[12] Bello-Ovosi BO, Ovosi JO, Ogunsina MA, Asuke S, Ibrahim MS (2019). Prevalence and pattern of dyslipidemia in patients with Type-2 diabetes mellitus in Zaria, Northwestern Nigeria. Pan Afr Med J.

[13] Katulanda P, Dissanayake HA, De Silva SDN, Katulanda GW, Liyanage IK, Constantine GR (2018). Prevalence, patterns, and associations of dyslipidemia among Sri Lankan adults-Sri Lanka Diabetes and Cardiovascular Study in 2005-2006. J Clin Lipidol.

[14] Das H, Banik S (2019). Prevalence of dyslipidemia among the diabetic patients in southern Bangladesh:A cross-sectional study. Diabetes Metab Syndr.

[15] Islam N, Rahman MZ, Choudhury S, Afrin L, Rahman S, Aftabuddin M (2012). Prevalence of Dyslipidemia and Associated Factors among the Sub-Urban Bangladeshi Population. Univ Heart J.

[16] Hussain A, Zakria M, Ali I, Tariq SA, Hussain A, Siraj S (2023). Pattern of dyslipidemia and associated factors in coronary artery disease patients in Khyber Pakhtunkhwa:A cross-sectional secondary data analysis. Pak J Med Sci.

[17] Ahmmed MS, Shuvo SD, Paul DK, Karim MR, Kamruzzaman M, Mahmud N (2021). Prevalence of dyslipidemia and associated risk factors among newly diagnosed Type-2 Diabetes Mellitus (T2DM) patients in Kushtia, Bangladesh. PLOS Glob Public Health.

[18] Li J, Nie Z, Ge Z, Shi L, Gao B, Yang Y (2022). Prevalence of dyslipidemia, treatment rate and its control among patients with Type-2 diabetes mellitus in Northwest China:a cross-sectional study. Lipids Health Dis.

[19] Huang R, Yan L, Lei Y (2021). The relationship between high density lipoprotein cholesterol (HDL-C) and glycosylated hemoglobin in diabetic patients aged 20 or above:a cross-sectional study. BMC Endocr disord.

[20] Hussain A, Ali I, Ijaz M, Rahim A (2017). Correlation between hemoglobin A1c and serum lipid profile in Afghani patients with Type-2 diabetes:hemoglobin A1c prognosticates dyslipidemia. Ther Adv Endocrinol Metab.

[21] Kidwai SS, Nageen A, Bashir F, Ara J (2020). HbA1c - A predictor of dyslipidemia in Type-2 Diabetes Mellitus. Pak J Med Sci.

[22] Begum A, Irfan SR, Hoque MR (2019). Relationship between HbA1c and lipid profile seen in Bangladeshi Type-2 Diabetes Mellitus patients attending BIRDEM Hospital:A cross-sectional study. Mymensingh Med J.

[23] Dana H (2022). Dyslipidemia among patients with Type-2 diabetes in Jorden:prevalence, pattern, and associated factors. Front Public Health.

[24] Khan HA, Sobki SH, Khan SA (2007). Association between glycaemic control and serum lipids profile in Type-2 diabetic patients:HbA1c predicts dyslipidaemia. Clin Exp Med.

[25] Hammad IK, Abed BA, Rashid NF (2012). Glycated Haemoglobin as a dual biomarker association between HbA1c and dyslipidemia in Type-2 diabetic patients. J Faculty Med Baghdad.

[26] Zikiria S, Hamid S (2019). Association of HTN and dyslipidemia with increasing obesity in pts with Type-2 DM. Braz J Pharm Sci.

[27] Narindrarangkura P, Bosl W, Rangsin R, Hatthachote P (2019). Prevalence of dyslipidemia associated with complications in diabetic patients:a nationwide study in Thailand. Lipids Health Dis.

[28] Mehta RK, Koirala P (2021). Dyslipidemia in patients with Type-2 diabetes in a tertiary care center:A descriptive cross- sectional study. J Nepal Med Assoc.

